# The Scramble conversion tool

**DOI:** 10.1093/bioinformatics/btu390

**Published:** 2014-06-14

**Authors:** James K. Bonfield

**Affiliations:** DNA Pipelines, Wellcome Trust Sanger Institute, Cambridgeshire, CB10 1SA, UK

## Abstract

**Motivation:** The reference CRAM file format implementation is in Java. We present ‘Scramble’: a new C implementation of SAM, BAM and CRAM file I/O.

**Results:** The C implementation of for CRAM is 1.5–1.7× slower than BAM at decoding but 1.8–2.6× faster at encoding. We see file size savings of 34–55%.

**Availability and implementation:** Source code is available at http://sourceforge.net/projects/staden/files/io_lib/ under the BSD software licence.

**Contact:** jkb@sanger.ac.uk

**Supplementary information**: Supplementary data are available at *Bioinformatics* online.

## 1 INTRODUCTION

Storage capacity has been the primary driver behind the development of the CRAM format (Cochrane *et al.*, 2013). The CRAM format (Fritz *et al.*, 2011) is a practical implementation of reference-based compression and is a viable alternative to the earlier BAM format (Li *et al.*, 2009). CRAM is now the preferred submission format for the European Nucleotide Archive.

The initial CRAM prototype was in Python, quickly followed by a Picard (http://picard.sourceforge.net/) compatible Java reference implementation (https://www.ebi.ac.uk/ena/about/cram_toolkit). We identified a need for a C implementation, which was implemented as part of the Staden Package’s (Staden *et al.*, 1999) ‘io_lib’ library.

Our primary conversion tool is named Scramble. It can read and write SAM, BAM and CRAM formats using a unified Application Programming Interface (API).

## 2 METHODS

We will not cover the CRAM file format here except to note that CRAM internally separates data by type before compressing with Zlib (Deutsch and Gailly, 1996). Thus, we have regular blocks of quality values, blocks of sequence names and blocks of auxiliary tags, each of which may be compressed using different Zlib parameters. A key efficiency observation is that using the run-length-encoding strategy (‘Z_RLE’) is considerably faster than the default strategy, while also often offering slightly higher compression ratios for quality values. It also allows for applications to potentially omit decoding of irrelevant data types. Note that these tricks are not possible in the BAM format, as all data types are interleaved within the same Zlib blocks.

Our implementation periodically samples both Z_RLE and the default strategy on data blocks to determine the optimal method. This ensures rapid speed without loss in compression ratio.

Multi-threading is implemented using a thread pool, shared by both encoding and decoding tasks. This contrasts well when compared with Samtools that can only parallelize file encoding. It also permits the most efficient use of threads when converting between differing file formats, automatically balancing the encoder and decoder work loads. Note that our SAM encoding and decoding is single threaded.

## 3 RESULTS AND DISCUSSION

We tested our implementation against the reference Java Cramtools implementation as well as existing BAM implementations in C (Samtools) and Java (Picard). The test data used were a 4× coverage of a *Homo sapiens* sample (ERR317482) aligned by BWA, with a further 1000 Genomes, and a 654× coverage *Escherichia coli* test set included in the Supplementary Material.

A breakdown of the file size by item type within the Scramble CRAM output can be seen in Table 1. The impact of lossy compression on quality values was also tested by applying Illumina’s quantizing system that portions the 40 distinct values into eight new bins (http://res.illumina.com/documents/products/whitepapers/whitepaper_datacompression.pdf). This reduced the file size by 39%; however, even in the reduced file the quality values still accounted for the bulk of the storage costs.

**Table 1.** CRAM breakdown by file percentage

Data type	File % age (40 Quality bins)	File % age (8 Quality bins)
Quality values	80.9	68.6
Sequence identifiers	8.3	13.7
Auxiliary tags	3.9	6.4
Flags	1.5	2.5
Alignment position	1.4	2.4
CIGAR string	1.4	2.3
Sequence bases	1.3	2.1
Template position/size	0.6	1.0
Mapping quality	0.2	0.4
Other/overhead	0.5	0.8

*Note*: Total file sizes for ERR317482: 3.46 Gb for 40 bins, 2.11 Gb for 8 bins.

Table 2 shows the time taken to read and write formats from the various tools along with their resultant file sizes. For encoding, it is clear that the C implementation of CRAM is considerably faster than the Java implementation and also beats Picard/Samtools BAM speed despite the use of the Intel-tuned Deflate implementation by Picard. This is almost entirely down to the use of Z_RLE for encoding quality values. Decoding of CRAM is not as fast as C BAM, but it is comparable with the widely used Picard’s BAM decoder. The nature of a column-oriented CRAM file allows for the *samtools flagstat* equivalent to run considerably faster. We also observe that the CRAM files produced by Scramble are around 9% smaller than those produced by Cramtools.jar.

**Table 2.** 9827_2#49.bam (ERR317482)

		40 quality bins					8 quality bins				
Tool	Format	Read(s)	Write(s)	Flagstat	Index	Size (Gb)	Read(s)	Write(s)	Flagstat	Index	Size (Gb)
Scramble	BAM	**76.9**	773.6	76.9	–	6.50	**63.3**	1063.6	63.3	–	4.80
Scramble	CRAM	117.1	**307.8**	**28.2**	**2.5**	**3.46**	111.1	**299.6**	**27.3**	**2.1**	**2.11**
Cramtools	CRAM	223.1	1333.2	–	48.4	3.78	209.0	1217.1	–	63.8	2.33
Samtools	BAM	89.1	759.0	89.1	81.1	6.50	69.6	1053.8	69.6	64.7	4.80
Picard	BAM	120.8	518.4	–	124.8	6.52	111.9	460.6	–	113.1	4.90

*Note*: User + System CPU times in seconds for encoding and decoding along with the produced file size. The timings correspond to a single 2.2 GHz Intel Xeon E5-2660 (of 16). The data were in the file system cache, and so these tasks are CPU-bound. Note that not all tools provide *index* and *flagstat* equivalents for all file formats, and so timings are omitted in these cases. Bold values represent the fastest or smallest figure in each column.

**Fig. 1.**
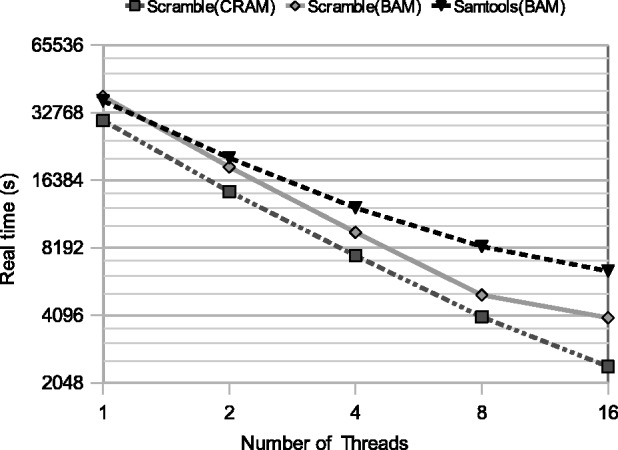
Real time taken to convert from 230 Gb BAM to BAM (Scramble, Samtools) and BAM to CRAM (Scramble) formats. The system was a 16 core 2.2 GHz Intel Xeon E5-2660 with a local RAID XFS file system. Tests on slower disks and with smaller locally cached data files are in the Supplementary Material, including benchmarks of Sambamba (https://github.com/lomereiter/sambamba) and Biobambam (Tischler and Leonard, 2013)

Scramble has full multi-threading support for both reading and writing of BAM and CRAM file formats. It scales nearly linearly up to 16 cores, but with some performance inefficiencies becoming visible in CRAM with high core counts, especially for decoding. The results for conversion timings can be seen in Figure 1.

## 4 CONCLUSION

We have demonstrated that the C implementation of CRAM performs well, beating Samtools, Picard and Cramtools for encoding speed. Decoding speed is not as efficient as Samtools but is still comparable with Picard and nearly twice as fast as the Java CRAM implementation. Also notable is that the nature of CRAM means some read operations (for example, *flagstat* and *index*) are faster than with BAM.

CRAM is not yet capable of achieving the top compression ratios, using 3.96 bits/base with 40 quality bins and 2.05 bits/base with 8 bins compared against only 3.16 and 1.52 for fqz_comp (Bonfield and Mahoney, 2013), and 41 bits per read name in CRAM versus 23 bits in fqz_comp. This demonstrates room for improvement in future CRAM versions, partially achieved by replacing Zlib with arithmetic coding or an Asymmetric Numerical System (Duda, 2013).

Scramble is not a drop-in replacement for the Samtools API; however, a port of the CRAM components of Scramble has been made to the HTSlib library and is available within Samtools version 1.0, available at https://github.com/samtools/.

## Supplementary Material

Supplementary Data
