# Supplementary material for: The Scramble conversion tool
Source: Bioinformatics. 2014 Jun 14;30(19):2818–9. doi: 10.1093/bioinformatics/btu390 (PMC4173023; doi:10.1093/bioinformatics/btu390)
Supplement: Supplementary Data [file supp_30_19_2818__index.html]

The Scramble Conversion Tool — The Scramble conversion tool — The Scramble conversion tool — Supplementary Data 

# The Scramble conversion tool

## Supplementary Data

files

**Files in this Data Supplement:**

- Supplementary Data - pdf file
