# Supplementary material for: The Scramble conversion tool
Source: Bioinformatics. 2014 Jun 14;30(19):2818–9. doi: 10.1093/bioinformatics/btu390 (PMC4173023; doi:10.1093/bioinformatics/btu390)
Supplement: Supplementary Data [file supp_btu390_Supplementary.pdf]

Supplementary Information for "The Scramble Conversion Tool"

Introduction

Due to size constraints the paper concentrates on the C CRAM implementation with a direct comparison to the reference Java implementation and the de-facto standard C/Java BAM implementations for context.

There are many other SAM/BAM implementations, some of which multi-thread better than Samtools. We compare against two such tools here for one of the multi-threading tests; Biobambam and Sambamba. This isn't intended to be an exhaustive review paper though.

To compute the separate READ vs WRITE timings was in some cases a challenge. Read times are mainly computed using *samtools flagstat* equivalents where applicable. For Picard we used the BAM indexer as this was the fastest tool to read BAM without having to write our own Java program. Neither of these are ideal, but typically the application part (flagstat, index) is of insignificant CPU time compared to the decompression and decoding. Cramtools is built on top of Picard so we use the Picard read timings as a proxy for Cramtools BAM decoding speed. Scramble's read timing on CRAM files is computed twice; once from the standard *scram_flagstat* tool and once with tool (derived from *scram_flagstat)* with the `CRAM_OPT_REQUIRED_FIELDS` line removed. The effect of this is to make the CRAM reader decode the entire CRAM file rather than only the fields necessary to produce the flagstat output, providing a more useful comparison of read speed.

WRITE timings in the paper are simply the time taken to perform a BAM->BAM or BAM->CRAM conversion with the BAM read timings subtracted.

Two test systems were used:

1) 16 core 2.2GHz Intel Xeon CPU E5-2660, with a 11TB local XFS raid array.
This was the default test system for all benchmarks unless specified otherwise.

2) 32 core 2.4GHz AMD Opteron Processor 6272, with a 1.0 TB local XFS disc.
A few tests are listed against this system. It served as a useful stress test for a larger thread count on a very restrictive I/O throughput system.

Program versions

Biobambam 0.0.129
Cramtools 2.1
Fqz_comp 4.6
Sambamba 0.4.6
Samtools 0.1.19
Scramble 1.13.7
Picard 1.113

ERR317482: Raw benchmark output for conversion timings (9827_2#49)

Based on http://www.ebi.ac.uk/ena/data/view/ERR317482. This is a submitted BAM file produced directly from alignment with BWA. The organism is Human. This test also includes a few SAM read/write speed tests, not included in the main paper. There are two READ CRAM timings with the second (*) being from *scram_flagstat* program. This skips decoding unnecessary fields, demonstrating one benefit of the CRAM format over BAM.

R=real time, C=CPU user time, S=CPU system time

```
=== 40-qual: 9827_2#49 ===
Scramble  READ BAM    R   77.29   C   75.35   S  1.56
Scramble  BAM2CRAM    R  389.64   C  372.15   S 12.56
Scramble  READ CRAM   R  133.17   C   99.47   S 17.62
Scramble  READ CRAM*  R   29.16   C   21.87   S  6.32
Scramble  BAM2BAM     R  857.24   C  834.62   S 15.88
Scramble  BAM2SAM     R  157.57   C  124.11   S 17.08
Scramble  SAM2SAM     R  124.46   C   79.26   S 28.47
Scramble  READ SAM    R   69.25   C   41.58   S 11.48

Samtools  READ BAM    R   89.15   C   80.36   S  1.54
Samtools  BAM2BAM     R  842.67   C  821.14   S 19.76
Samtools  BAM2SAM     R  170.77   C  149.15   S 21.22
Samtools  SAM2SAM     R  252.63   C  206.18   S 31.28

Cramtools READ BAM    (See Picard)
Cramtools BAM2CRAM    R 1342.47   C 1443.56   S 16.12
Cramtools READ CRAM   R  207.89   C  219.27   S  3.86

Picard    READ BAM    R  120.83   C  123.15   S  3.35
Picard    BAM2BAM     R  641.97   C  637.50   S  7.36
Picard    BAM2SAM     R  416.80   C  406.78   S 18.85
Picard    SAM2SAM     R  494.79   C  471.29   S 31.34

Sambamba  READ BAM    R   88.44   C   83.29   S  1.62
Sambamba  BAM2BAM     R  910.53   C  889.65   S 18.70

=== 8-qual: 9827_2#49 ===
Scramble  READ BAM    R   63.52   C   62.42   S  0.91
Scramble  BAM2CRAM    R  363.66   C  354.73   S  8.22
Scramble  READ CRAM   R  120.73   C   93.31   S 17.84
Scramble  READ CRAM*  R   27.45   C   21.60   S  5.70
Scramble  BAM2BAM     R 1128.79   C 1114.02   S 12.91
Scramble  BAM2SAM     R  128.20   C   99.91   S 15.95
Scramble  SAM2SAM     R  138.74   C   75.26   S 28.79
Scramble  READ SAM    R   55.01   C   41.50   S  9.80

Samtools  READ BAM    R   75.29   C   68.36   S  1.21
Samtools  BAM2BAM     R 1126.71   C 1106.78   S 16.59
Samtools  BAM2SAM     R  157.50   C  137.27   S 19.44
Samtools  SAM2SAM     R  287.92   C  219.96   S 31.22

Cramtools READ BAM    (See Picard)
Cramtools BAM2CRAM    R 1356.14   C 1452.52   S 15.74
Cramtools READ CRAM   R  191.67   C  205.50   S  3.47

Picard    READ BAM    R  106.20   C  108.99   S  2.87
Picard    BAM2BAM     R  569.56   C  566.67   S  5.82
Picard    BAM2SAM     R  432.73   C  412.69   S 18.85
Picard    SAM2SAM     R  514.26   C  477.60   S 30.72

Sambamba  READ BAM    R   82.41   C   71.65   S  1.43
Sambamba  BAM2BAM     R 1181.30   C 1158.25   S 17.95
```

(*) Timing from scram_flagstat

File sizes for ERR317482

```
Q40
-rw-r--r-- 1 jkb team117  3778328074 May 19 13:59 9827_2#49.java.cram
-rw-r--r-- 1 jkb team117  6517466114 May 19 14:24 9827_2#49.picard.bam
-rw-r--r-- 1 jkb team117 21058600749 May 19 13:21 9827_2#49.sam
-rw-r--r-- 1 jkb team117  6499958741 May 19 15:10 9827_2#49.sambamba.bam
-rw-r--r-- 1 jkb team117  6499959405 May 19 13:17 9827_2#49.samtools.bam
-rw-r--r-- 1 jkb team117  6499339396 May 19 12:49 9827_2#49.scramble.bam
-rw-r--r-- 1 jkb team117  3456826779 May 19 12:27 9827_2#49.scramble.cram

Q8
-rw-r--r-- 1 jkb team117  2322148894 May 19 17:01 9827_2#49.bin.java.cram
-rw-r--r-- 1 jkb team117  4896592994 May 19 17:24 9827_2#49.bin.picard.bam
-rw-r--r-- 1 jkb team117 21058600885 May 19 16:25 9827_2#49.bin.sam
-rw-r--r-- 1 jkb team117  4804359822 May 19 18:14 9827_2#49.bin.sambamba.bam
-rw-r--r-- 1 jkb team117  4804360472 May 19 16:21 9827_2#49.bin.samtools.bam
-rw-r--r-- 1 jkb team117  4803961451 May 19 15:50 9827_2#49.bin.scramble.bam
-rw-r--r-- 1 jkb team117  2105509056 May 19 15:24 9827_2#49.bin.scramble.cram
```

Size breakdowns for ERR317482

Three letter IDs such as "AMc" and "XCc" are auxiliary tags. Two letter ones correspond to the CRAM data series.

```
Q40:  cram_dump 9827_2#49.scramble.cram | tail -26
Block CORE          , total size    9913862
Block content_id   1, total size   45927981  AMc XCc X0c X1C XOc X0C X1c SMc X0S X1S ahc XMc XGc a3c XTA X0s XNc X1s
Block content_id   6, total size   62717282  QTZ
Block content_id   7, total size    4325412  BCZ
Block content_id   8, total size   21327985  XAZ
Block content_id  10, total size  287857275  RN
Block content_id  11, total size 2795090929  QS
Block content_id  12, total size    5274813  SC
Block content_id  13, total size     494650  IN
Block content_id  14, total size   27256424  BF
Block content_id  15, total size   10759295  CF
Block content_id  16, total size   49946088  AP
Block content_id  18, total size    7474882  MQ
Block content_id  19, total size     524578  NS
Block content_id  20, total size     534542  MF
Block content_id  21, total size    2502227  TS
Block content_id  22, total size    4902317  NP
Block content_id  23, total size   13982587  NF
Block content_id  25, total size   12913131  FN
Block content_id  26, total size    4144426  FC
Block content_id  27, total size   30511457  FP
Block content_id  28, total size      57092  DL
Block content_id  29, total size   28585274  BA
Block content_id  30, total size    8987964  BS
Block content_id  31, total size   13275634  TL
Block content_id  32, total size       7434  RI
```

Q8: cram_dump 9827_2#49.bin.scramble.cram | tail -26

```
Block CORE          , total size    9913862
Block content_id   1, total size   45927981  AMc XCc X0c X1C XOc X0C X1c SMc X0S X1S ahc
XMc XGc a3c XTA X0s XNc X1s
Block content_id   6, total size   62717282  QTZ
Block content_id   7, total size    4325412  BCZ
Block content_id   8, total size   21327985  XAZ
Block content_id  10, total size  287857275  RN
Block content_id  11, total size 1443773000  QS
Block content_id  12, total size    5274813  SC
Block content_id  13, total size     504360  IN
Block content_id  14, total size   27256424  BF
Block content_id  15, total size   10759295  CF
Block content_id  16, total size   49946088  AP
Block content_id  18, total size    7474882  MQ
Block content_id  19, total size     524580  NS
Block content_id  20, total size     534542  MF
Block content_id  21, total size    2502227  TS
Block content_id  22, total size    4902317  NP
Block content_id  23, total size   13982587  NF
Block content_id  25, total size   12913131  FN
Block content_id  26, total size    4144426  FC
Block content_id  27, total size   30511457  FP
Block content_id  28, total size      57092  DL
Block content_id  29, total size   28585274  BA
Block content_id  30, total size    8987964  BS
Block content_id  31, total size   13275634  TL
Block content_id  32, total size       7434  RI
```

Fastq compression metrics

Quality encoding tested with fqz_comp. This uses probabilistic modelling and arithmetic coding. It is not realistic to expect CRAM to compete given CRAM requires random access and compresses each 10000 reads independently, however it gives a useful baseline to compare against.

```
$ fqz_comp < 9827_2#49.fastq > /dev/null
Names 1891639400 ->  165489689 (0.087)
Bases 5646323600 -> 1213484148 (0.215)
Quals 5646323600 -> 2456779729 (0.435)

$ fqz_comp < 9827_2#49.bin.fastq > /dev/null
Names 1891639400 ->  165489689 (0.087)
Bases 5646323600 -> 1213484148 (0.215)
Quals 5646323600 -> 1097053253 (0.194)

$ fqz_comp -n2 -s6+ -q3 < 9827_2#49.fastq > /dev/null
Names 1891639400 ->  165489689 (0.087)
Bases 5646323600 -> 1171170282 (0.207)
Quals 5646323600 -> 2228676073 (0.395)

$ fqz_comp -n2 -s6+ -q3 < 9827_2#49.bin.fastq > /dev/null
Names 1891639400 ->  165489689 (0.087)
Bases 5646323600 -> 1182521177 (0.209)
Quals 5646323600 -> 1071594874 (0.190)
```

ERR251692: Raw benchmark output for conversion timings (NA21144.chrom11)

We used the BAM file produced by the 1000 Genomes consortium, chosen simply because it was the last individual.

ftp://ftp.1000genomes.ebi.ac.uk/vol1/ftp/data/NA21144/alignment/NA21144.chrom11.ILLUMINA.bwa.GIH.low_coverage.20130415.bam

The data set differs to the above one in that it has been processed by GATK. This means the quality values have higher entropy due to the recalibration process and it now has additional auxiliary tags, specifically a BQ:Z: string to hold the base alignment qualities. The impact is slightly reduced CRAM performance.

```
=== 40-qual: NA21144.chrom11.ILLUMINA.bwa.GIH.low_coverage.20130415 ===
Scramble  READ BAM    R   13.10   C   12.59    S  0.27
Scramble  BAM2CRAM    R   66.73   C   64.06    S  2.50
Scramble  READ CRAM   R   21.35   C   18.00    S  2.76
Scramble  READ CRAM*  R    4.22   C    3.25    S  0.91
Scramble  BAM2BAM     R  132.13   C  129.23    S  2.60
Scramble  BAM2SAM     R   27.60   C   23.98    S  3.53
Scramble  SAM2SAM     R   20.81   C   16.08    S  4.67
Scramble  READ SAM    R   7.66    C    6.86    S  0.79

Samtools  READ BAM    R   12.90   C   12.67    S  0.18
Samtools  BAM2BAM     R  128.89   C  125.11    S  3.56
Samtools  BAM2SAM     R   34.22   C   29.21    S  4.94
Samtools  SAM2SAM     R  42.37    C   36.27    S  5.96

Cramtools READ BAM    (See Picard)
Cramtools BAM2CRAM    R  257.72   C  273.00    S  5.13
Cramtools READ CRAM   R   41.89   C   45.57    S  1.20

Picard    READ BAM    R   22.49   C   22.84    S  0.55
Picard    BAM2BAM     R  105.82   C  105.63    S  1.21
Picard    BAM2SAM     R   74.38   C   72.81    S  4.16
Picard    SAM2SAM     R   80.03   C   76.64    S  5.86

===  8-qual: NA21144.chrom11.ILLUMINA.bwa.GIH.low_coverage.20130415 ===
Scramble  READ BAM    R   10.92   C   10.65   S  0.16
Scramble  BAM2CRAM    R   63.77   C   61.42   S  2.12
Scramble  READ CRAM   R   20.80   C   17.86   S  2.30
Scramble  READ CRAM*  R    3.99   C    3.07   S  0.88
Scramble  BAM2BAM     R  210.96   C  207.95   S  2.67
Scramble  BAM2SAM     R   18.68   C   15.53   S  3.10
Scramble  SAM2SAM     R   21.77   C   16.10   S  4.72
Scramble  READ SAM    R    8.47   C    6.88   S  0.81

Samtools  READ BAM    R   10.90   C   10.63   S  0.20
Samtools  BAM2BAM     R  208.03   C  204.57   S  3.11
Samtools  BAM2SAM     R   31.36   C   26.07   S  5.24
Samtools  SAM2SAM     R   41.64   C   36.06   S  5.52

Cramtools READ BAM    (See Picard)
Cramtools BAM2CRAM    R  267.69   C  285.82   S  4.88
Cramtools READ CRAM   R   39.14   C   43.02   S  1.04

Picard    READ BAM    R   18.74   C   19.31   S  0.53
Picard    BAM2BAM     R   95.58   C   95.47   S  0.86
Picard    BAM2SAM     R   69.97   C   68.85   S  3.70
Picard    SAM2SAM     R   83.03   C   79.09   S  6.30
```

File sizes

Q40
```
 665219874 NA21144.chrom11.ILLUMINA.bwa.GIH.low_coverage.20130415.java.cram
1047474221 NA21144.chrom11.ILLUMINA.bwa.GIH.low_coverage.20130415.picard.bam
4504400097 NA21144.chrom11.ILLUMINA.bwa.GIH.low_coverage.20130415.sam
1050955355 NA21144.chrom11.ILLUMINA.bwa.GIH.low_coverage.20130415.samtools.bam
1050828610 NA21144.chrom11.ILLUMINA.bwa.GIH.low_coverage.20130415.scramble.bam
 621760172 NA21144.chrom11.ILLUMINA.bwa.GIH.low_coverage.20130415.scramble.cram
```

Q8
```
 401710252 NA21144.chrom11.ILLUMINA.bwa.GIH.low_coverage.20130415.bin.java.cram
 752295317 NA21144.chrom11.ILLUMINA.bwa.GIH.low_coverage.20130415.bin.picard.bam
4504400547 NA21144.chrom11.ILLUMINA.bwa.GIH.low_coverage.20130415.bin.sam
 742158126 NA21144.chrom11.ILLUMINA.bwa.GIH.low_coverage.20130415.bin.samtools.bam
 742074691 NA21144.chrom11.ILLUMINA.bwa.GIH.low_coverage.20130415.bin.scramble.bam
 367805198 NA21144.chrom11.ILLUMINA.bwa.GIH.low_coverage.20130415.bin.scramble.cram
```

The differing file sizes for BAM output mainly come from using the libIntelDeflator.so with Picard, which uses a (usually) lighter weight compression algorithm.

Size breakdowns for ERR251692 (1000Genomes alignment)

Q40
```
$ cram_dump NA21144.chrom11.ILLUMINA.bwa.GIH.low_coverage.20130415.scramble.cram | tail
Block CORE          , total size      20090
Block content_id   1, total size    6495529  AMc XCc X0c X1i X1C X0C MQc X1c SMc X0S OPi
X0i X1S XTA X0s XNc X1s
Block content_id   3, total size   33068235  BQZ
Block content_id   8, total size    1697855  XAZ OCZ
Block content_id  10, total size   35439079  RN
Block content_id  11, total size  503518090  QS
Block content_id  12, total size    9116842  SC
Block content_id  13, total size      59262  IN
Block content_id  14, total size    4964866  BF
Block content_id  15, total size    1896325  CF
Block content_id  16, total size    6673783  AP
Block content_id  17, total size    1857380  RG
Block content_id  18, total size    1020561  MQ
Block content_id  19, total size      91219  NS
Block content_id  20, total size      47394  MF
Block content_id  21, total size     116288  TS
Block content_id  22, total size     345958  NP
Block content_id  23, total size    3130686  NF
Block content_id  25, total size    2163656  FN
Block content_id  26, total size     978981  FC
Block content_id  27, total size    3997027  FP
Block content_id  28, total size         80  DL
Block content_id  29, total size    1163976  BA
Block content_id  30, total size     929661  BS
Block content_id  31, total size    1645333  TL
```

Q8
```
$ cram_dump NA21144.chrom11.ILLUMINA.bwa.GIH.low_coverage.20130415.bin.scramble.cram |
tail|grep QS
Block content_id  11, total size  249562422  QS
```

```
(The remainder of the CRAM data series elements are the same size as the Q40
test.)
```

ERR376619: Raw benchmark output for conversion timings (9799_7#3)

Based on http://www.ebi.ac.uk/ena/data/view/ERR376619. This is a submitted BAM file produced directly from alignment with BWA. The organism is E.Coli and this test represents deeply sequenced (654x) data for a non-mammalian genome.

```
=== 40-qual: 9799_7#3 ===
Scramble  READ BAM    R   35.10   C   32.44   S  0.77
Scramble  BAM2CRAM    R  190.17   C  184.56   S  5.04
Scramble  READ CRAM   R   51.30   C   44.86   S  5.31
Scramble  READ CRAM*  R    9.63   C    7.49   S  2.06
Scramble  BAM2BAM     R  498.19   C  490.62   S  6.76
Scramble  BAM2SAM     R   59.88   C   51.54   S  8.20
Scramble  SAM2SAM     R   82.28   C   44.69   S 14.35
Scramble  READ SAM    R   46.96   C   26.49   S  3.13

Samtools  READ BAM    R   34.75   C   32.83   S  0.57
Samtools  BAM2BAM     R  485.54   C  476.50   S  8.21
Samtools  BAM2SAM     R   83.58   C   71.93   S 11.44
Samtools  SAM2SAM     R  119.58   C  103.43   S 15.01

Cramtools READ BAM    (See Picard)
Cramtools BAM2CRAM    R  700.44   C  765.07   S  7.06
Cramtools READ CRAM   R  106.40   C  112.29   S  2.34

Picard    READ BAM    R   54.66   C   55.49   S  0.96
Picard    BAM2BAM     R  296.72   C  295.18   S  2.62
Picard    BAM2SAM     R  203.47   C  198.23   S 10.48
Picard    SAM2SAM     R  257.95   C  244.41   S 14.10

===  8-qual: 9799_7#3 ===
Scramble  READ BAM    R   24.08   C   23.75   S  0.25
Scramble  BAM2CRAM    R  166.46   C  162.33   S  3.78
Scramble  READ CRAM   R   47.67   C   41.94   S  5.36
Scramble  READ CRAM*  R    9.46   C    7.41   S  2.00
Scramble  BAM2BAM     R  398.26   C  392.10   S  5.49
Scramble  BAM2SAM     R   45.00   C   37.46   S  7.41
Scramble  SAM2SAM     R   89.05   C   38.40   S 12.65
Scramble  READ SAM    R   41.41   C   17.13   S  2.12

Samtools  READ BAM    R   29.30   C   24.72   S  0.43
Samtools  BAM2BAM     R  396.63   C  388.19   S  7.39
Samtools  BAM2SAM     R   75.71   C   63.38   S 12.11
Samtools  SAM2SAM     R  118.53   C  101.10   S 14.96

Cramtools READ BAM    (See Picard)
Cramtools BAM2CRAM    R  587.82   C  642.94   S  5.94
Cramtools READ CRAM   R   94.91   C  100.98   S  1.98

Picard    READ BAM    R   44.24   C   44.96   S  0.77
Picard    BAM2BAM     R  234.30   C  233.75   S  1.82
Picard    BAM2SAM     R  189.05   C  183.59   S  9.83
Picard    SAM2SAM     R  248.73   C  241.37   S 13.82
```

File sizes

Q40

```
 1776898862 9799_7#3.java.cram
 2459845559 9799_7#3.picard.bam
11538417497 9799_7#3.sam
 2438766221 9799_7#3.samtools.bam
 2438649501 9799_7#3.scramble.bam
 1609470501 9799_7#3.scramble.cram
```

Q8

```
  981052794 9799_7#3.bin.java.cram
 1559851113 9799_7#3.bin.picard.bam
11538417625 9799_7#3.bin.sam
 1528268039 9799_7#3.bin.samtools.bam
 1528192421 9799_7#3.bin.scramble.bam
  899244915 9799_7#3.bin.scramble.cram
```

Size breakdowns

Q40

```
$ ~/io_lib/build.seq3/progs/cram_dump 9799_7#3.cram |tail -25
Block CORE          , total size      13329
Block content_id   1, total size   10872475  AMc XCc X0c XOc X1c SMc ahc XMc XGc a3c XTA
Block content_id   6, total size   19111484  QTZ
Block content_id   7, total size     919313  BCZ
Block content_id   8, total size    2706216  XAZ
Block content_id  10, total size  200698904  RN
Block content_id  11, total size 1248538783  QS
Block content_id  12, total size    1243670  SC
Block content_id  13, total size      53773  IN
Block content_id  14, total size   19571505  BF
Block content_id  15, total size    6430079  CF
Block content_id  16, total size    4387722  AP
Block content_id  18, total size    4141507  MQ
Block content_id  20, total size    1319431  MF
Block content_id  21, total size   24097182  TS
Block content_id  22, total size   23449130  NP
Block content_id  23, total size   17965455  NF
Block content_id  25, total size    4581765  FN
Block content_id  26, total size     618135  FC
Block content_id  27, total size    6019720  FP
Block content_id  28, total size         34  DL
Block content_id  29, total size    3035540  BA
Block content_id  30, total size    1665491  BS
Block content_id  31, total size    5554767  TL
```

Q8

```
$ ~/io_lib/build.seq3/progs/cram_dump 9799_7#3.cram |tail -25|grep QS
Block content_id  11, total size  538312999  QS
```

Multi-threaded timings for BAM → BAM and BAM → CRAM (ERR317482)

Tested on 16 core 2.4GHz Intel Xeon CPU E5-2660

Number of threads (16 to 1); Real, CPU and System times.
Two runs per test to check reproducability on a "live" system.

Here we also included Sambamba (http://lomereiter.github.io/sambamba/) and Biobambam (https://github.com/gt1/biobambam) as demonstrations of more favourable implementations of multi-threaded BAM reading and writing, albeit on one of the smaller test sets. Biobambam and Scramble as so close the lines are superimposed except for the final 16 thread test. Note that Biobambam's recompression tool is manipulating BGZF blocks rather than SAM/BAM records. Sambamba also shows good linear speed improvements, albeit consistently slightly slower.

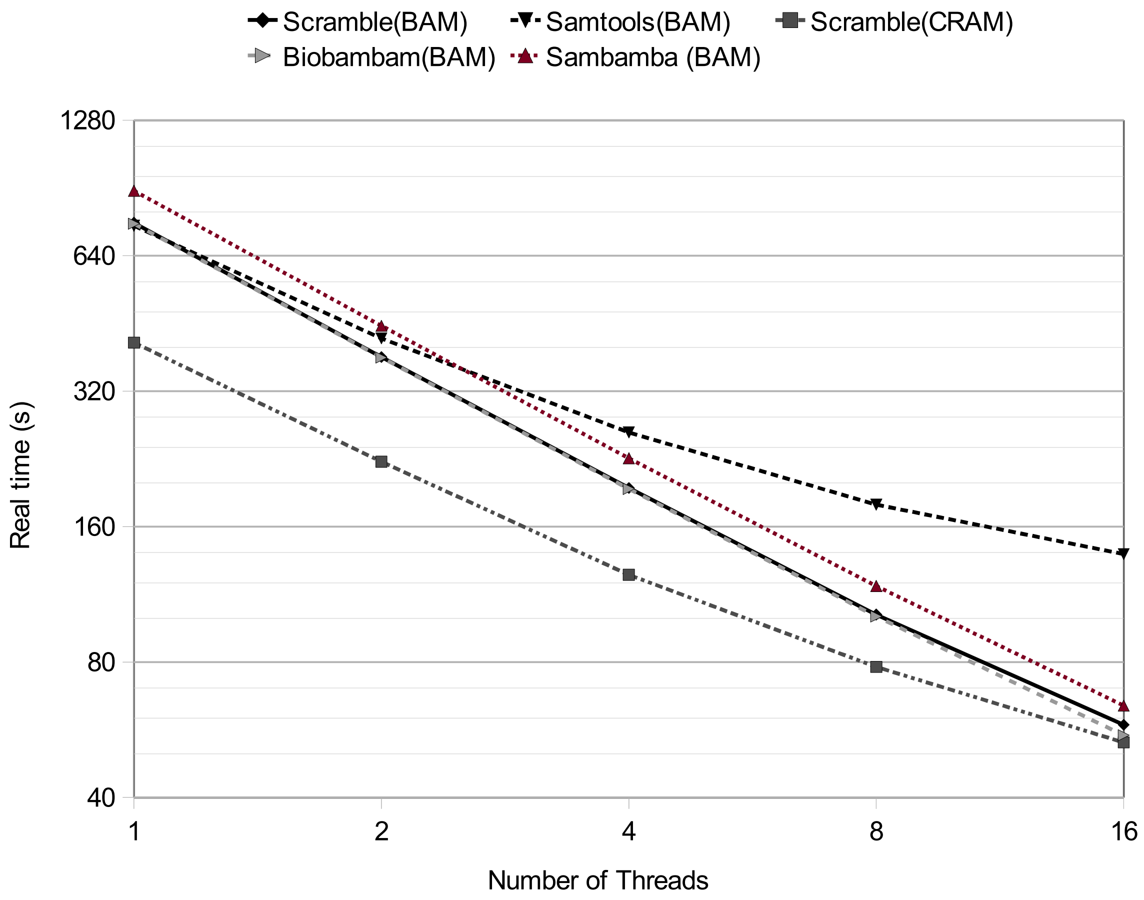

Samtools BAM → BAM

16	R 139.29	C 824.79	S 21.78
16	R 138.54	C 826.24	S 22.32
16	R 138.82	C 824.25	S 23.03
8	R 179.87	C 777.85	S 19.73
8	R 179.38	C 778.15	S 19.90
8	R 179.24	C 777.44	S 19.03
4	R 259.35	C 743.08	S 18.43
4	R 259.15	C 743.41	S 17.63
2	R 422.41	C 735.03	S 17.01
2	R 418.73	C 729.95	S 16.82
1	R 747.68	C 728.98	S 17.40
1	R 746.22	C 727.91	S 17.05

Biobambam BAM → BAM

16	R 54.94	C 835.70	S 8.74
16	R 54.71	C 834.36	S 9.56
16	R 54.57	C 837.36	S 9.68
8	R 101.14	C 796.73	S 7.84
8	R 101.10	C 796.11	S 8.19
8	R 101.01	C 794.62	S 8.93
4	R 197.49	C 782.06	S 8.05
4	R 193.75	C 767.23	S 8.05
2	R 380.01	C 755.09	S 7.81
2	R 379.61	C 753.84	S 8.13
1	R 753.91	C 748.19	S 8.69
1	R 753.70	C 747.42	S 8.78

Sambamba BAM → BAM

16	R 63.74	C 985.27	S 20.49
16	R 64.41	C 982.39	S 21.81
16	R 64.43	C 981.73	S 21.21
8	R 117.76	C 913.04	S 19.65
8	R 118.58	C 918.98	S 20.13
8	R 118.55	C 916.95	S 19.89
4	R 228.44	C 890.82	S 18.23
4	R 227.00	C 885.19	S 18.05
2	R 447.56	C 874.29	S 17.79
2	R 447.74	C 875.29	S 17.46
1	R 892.57	C 873.37	S 17.62
1	R 892.37	C 873.40	S 17.40

Scramble BAM → BAM

16	R 58.34	C 850.16	S 23.91
16	R 58.38	C 848.27	S 25.22
16	R 58.29	C 851.03	S 24.48
8	R 102.24	C 806.96	S 19.46
8	R 102.17	C 807.92	S 19.54
8	R 102.79	C 812.40	S 20.17
4	R 195.46	C 777.45	S 18.80
4	R 195.75	C 778.82	S 18.37
2	R 382.23	C 763.47	S 17.37
2	R 381.89	C 762.12	S 17.93
1	R 757.41	C 741.24	S 14.95
1	R 757.95	C 741.63	S 15.09

Scramble BAM → CRAM

16	R 52.93	C 512.14	S 23.51
16	R 58.92	C 512.16	S 23.91
16	R 61.62	C 513.38	S 24.34
8	R 77.30	C 464.80	S 21.35
8	R 79.45	C 467.29	S 21.88
8	R 77.51	C 464.26	S 20.06
4	R 124.81	C 426.98	S 17.54
4	R 124.98	C 428.82	S 18.91
2	R 222.62	C 420.77	S 15.81
2	R 225.38	C 425.07	S 15.95
1	R 410.20	C 384.18	S 12.52
1	R 409.78	C 383.36	S 13.04

Large file multi-threaded timing for converting BAM → BAM and BAM → CRAM

To test the impact of potentially I/O bound situations we tested using a 233Gb BAM file on the same machine used for other tests (which has 48Gb of memory) along with a system with a smaller, slower, disk.kilrr The BAM file can be obtained from:

ftp://ftp.1000genomes.ebi.ac.uk/vol1/ftp/data/NA12878/high_coverage_alignment/NA12878.mapped.ILLUMINA.bwa.CEU.high_coverage_pcr_free.20130906.bam

Data was read from and written to the same local XFS partition. With high number of threads this test becomes considerably I/O bound, designed to demonstrate the performance scaling when CPU is no longer the bottleneck. We tested on a system comprising a 16 core Intel CPU and a 11TB local RAID array formatted as XFS, as well as a rather more constrained 32 core AMD system with only a 1TB non-RAID XFS local disk. The CPU differences are largely irrelevant and simply reflect the test machines available, but the fast / slow disk I/O demonstrates how the tools behave when stressed.

The columns are number of threads, tool (CRAM(s) being the slower entire-decode of CRAM and CRAM(f) being the faster *scram_flagstat* tool), and the real, user-cpu and system-cpu times in seconds. We ran each test multiple times, especially the faster ones using more threads. We saw considerable variation in reading speed from CRAM, but little variability in the read/write tests.

The file sizes were:

```
250863909273  NA12878.mapped.ILLUMINA.bwa.CEU.high_coverage_pcr_free.20130906.bam
169035368370  NA12878.mapped.ILLUMINA.bwa.CEU.high_coverage_pcr_free.20130906.cram

Block CORE          , total size     8899416
Block content_id   1, total size  1360060636  XSc OPs MQc ASc OPS OPi ASC XSC
Block content_id   3, total size 16842849602  BQZ
Block content_id   4, total size 74980578237  BIZ BDZ
Block content_id   8, total size   252861290  SAZ OCZ
Block content_id  10, total size  4316355045  RN
Block content_id  11, total size 59786138298  QS
Block content_id  12, total size  5506733328  SC
Block content_id  13, total size    10061567  IN
Block content_id  14, total size   379027652  BF
Block content_id  15, total size   143163226  CF
Block content_id  16, total size   330438620  AP
Block content_id  17, total size   171714460  RG
Block content_id  18, total size    66838408  MQ
Block content_id  19, total size    10157822  NS
Block content_id  20, total size     7242183  MF
Block content_id  21, total size    88325602  TS
Block content_id  22, total size   129287260  NP
Block content_id  23, total size   330839164  NF
Block content_id  24, total size   317198804  RL
Block content_id  25, total size   321049768  FN
Block content_id  26, total size   382902248  FC
Block content_id  27, total size  2085604500  FP
Block content_id  28, total size     3990624  DL
Block content_id  29, total size   514714006  BA
Block content_id  30, total size   506778071  BS
Block content_id  31, total size    91813634  TL
Block content_id  35, total size     8104671  HC
```

Note that the BQ:Z, BI:Z and BD:Z auxiliary tags in this file consumed 54% of the title file size. This indicates that research also needs to be undertaken into the effect of quantising these quality values into 8 bins, in addition to the standard QS data series.

Read timings, slow disk I/O:

Clearly visible is the switch point where reading BAM becomes slower than reading CRAM due to the disk I/O becoming the bottleneck and the smaller CRAM being faster to read. Samtools here is represented as a flat line as there is no multi-threaded decoding. CRAM decoding speeds with large thread counts became rather variable.

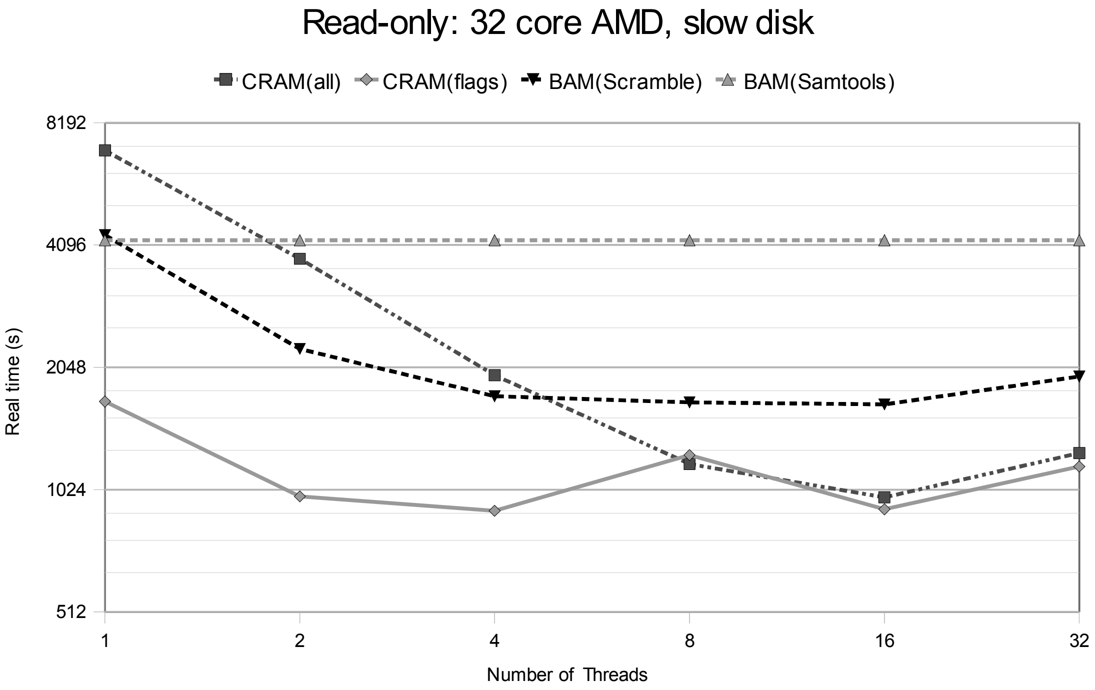


```
=== Scram_flagstat ===
32      CRAM(s) R 1376.28       C 8880.61       S 655.77
32      BAM     R 1632.87       C 9077.90       S 322.20
32      CRAM(f) R 1190.54       C 1669.93       S 554.72

32      CRAM(s) R 1224.74       C 9156.92       S 573.51
32      BAM     R 1635.85       C 9114.91       S 333.66
32      CRAM(f) R 1499.18       C 1590.26       S 440.70

32      CRAM(s) R 1597.56       C 8898.98       S 709.93
32      BAM     R 1643.50       C 9090.69       S 301.43
32      CRAM(f) R 1628.12       C 1743.05       S 541.41

32      CRAM(s) R 1109.98       C 8999.93       S 483.46
32      BAM     R 1654.67       C 9091.89       S 312.83
32      CRAM(f) R 890.83        C 1549.26       S 429.81

32      CRAM(s) R 1283.66       C 8930.41       S 679.57
32      BAM     R 1724.20       C 9055.24       S 325.48
32      CRAM(f) R 857.24        C 1581.46       S 389.40

32      CRAM(s) R 966.95        C 9052.24       S 416.77
32      BAM     R 1734.96       C 9121.93       S 318.86
32      CRAM(f) R 941.47        C 1615.43       S 301.86

16      CRAM(s) R 1127.61       C 8890.65       S 443.15
16      BAM     R 1635.88       C 9046.77       S 326.41
16      CRAM(f) R 892.90        C 1446.80       S 273.71

16      CRAM(s) R 990.94        C 8947.98       S 438.60
16      BAM     R 1636.69       C 9049.87       S 326.80
16      CRAM(f) R 840.84        C 1454.14       S 272.15
```

```
16      CRAM(s) R 961.22        C 8939.60       S 425.01
16      BAM     R 1641.47       C 9017.03       S 324.94
16      CRAM(f) R 903.17        C 1476.04       S 259.98

16      CRAM(s) R 986.58        C 8991.05       S 422.84
16      BAM     R 1638.76       C 9072.48       S 322.93
16      CRAM(f) R 835.91        C 1474.68       S 250.94

16      CRAM(s) R 912.86        C 8959.46       S 436.29
16      BAM     R 1716.58       C 9043.20       S 313.60
16      CRAM(f) R 879.50        C 1564.32       S 274.05

16      CRAM(s) R 902.67        C 8957.50       S 425.64
16      BAM     R 1692.98       C 9038.22       S 325.08
16      CRAM(f) R 1145.12       C 1593.72       S 287.94

8       CRAM(s) R 1205.62       C 8500.03       S 684.63
8       BAM     R 1634.84       C 9104.01       S 324.55
8       CRAM(f) R 1152.20       C 1573.50       S 327.21

8       CRAM(s) R 1208.95       C 8481.66       S 709.30
8       BAM     R 1738.69       C 9082.44       S 326.79
8       CRAM(f) R 1352.46       C 1542.34       S 287.63

8       CRAM(s) R 1110.74       C 8516.18       S 790.81
8       BAM     R 1638.65       C 9078.38       S 318.33
8       CRAM(f) R 896.13        C 1573.41       S 317.43

8       CRAM(s) R 1133.71       C 8387.83       S 759.26
8       BAM     R 1642.50       C 9011.86       S 320.70
8       CRAM(f) R 1064.03       C 1556.58       S 317.75

8       CRAM(s) R 1232.11       C 8490.83       S 685.19
8       BAM     R 1700.46       C 9116.03       S 264.95
8       CRAM(f) R 1506.94       C 1694.17       S 303.28

8       CRAM(s) R 1215.13       C 8651.20       S 731.41
8       BAM     R 1716.63       C 9072.14       S 289.74
8       CRAM(f) R 1506.12       C 1696.49       S 309.16

4       CRAM(s) R 1961.00       C 7727.86       S 1014.90
4       BAM     R 1721.89       C 5565.44       S 326.11
4       CRAM(f) R 982.99        C 1531.38       S 435.75

4       CRAM(s) R 1957.11       C 7738.23       S 1014.46
4       BAM     R 1756.97       C 5647.34       S 327.87
4       CRAM(f) R 833.58        C 1427.41       S 422.94

2       CRAM(s) R 3776.21       C 7630.01       S 1038.78
2       BAM     R 2272.18       C 4368.77       S 334.20
2       CRAM(f) R 1046.38       C 1336.13       S 530.08

2       CRAM(s) R 3802.37       C 7570.22       S 1082.36
2       BAM     R 2274.37       C 4369.58       S 328.28
2       CRAM(f) R 924.47        C 1247.10       S 525.71

1       CRAM(s) R 7046.93       C 6759.97       S 120.82
1       BAM     R 4286.67       C 3642.80       S 134.60
1       CRAM(f) R 1636.23       C 854.22        S 518.00

1       CRAM(s) R 6966.47       C 6802.89       S 115.39
1       BAM     R 4379.73       C 3638.96       S 146.57
1       CRAM(f) R 1737.92       C 918.32        S 535.17

=== Samtools flagstat BAM ===
1       BAM     R 4204.12       C 4027.78       S 168.72

1       BAM     R 4216.67       C 4024.49       S 182.04
```

Read/write timings, slow disk I/O:

The Scramble BAM → CRAM conversion slightly beats the Scramble BAM → BAM conversion with all numbers of threads, but shows some slow down at high thread counts. Samtools BAM → BAM conversion is comparable with 1 thread but the lack of multi-threaded decoding means it slows down considerably as the number of threads increases.

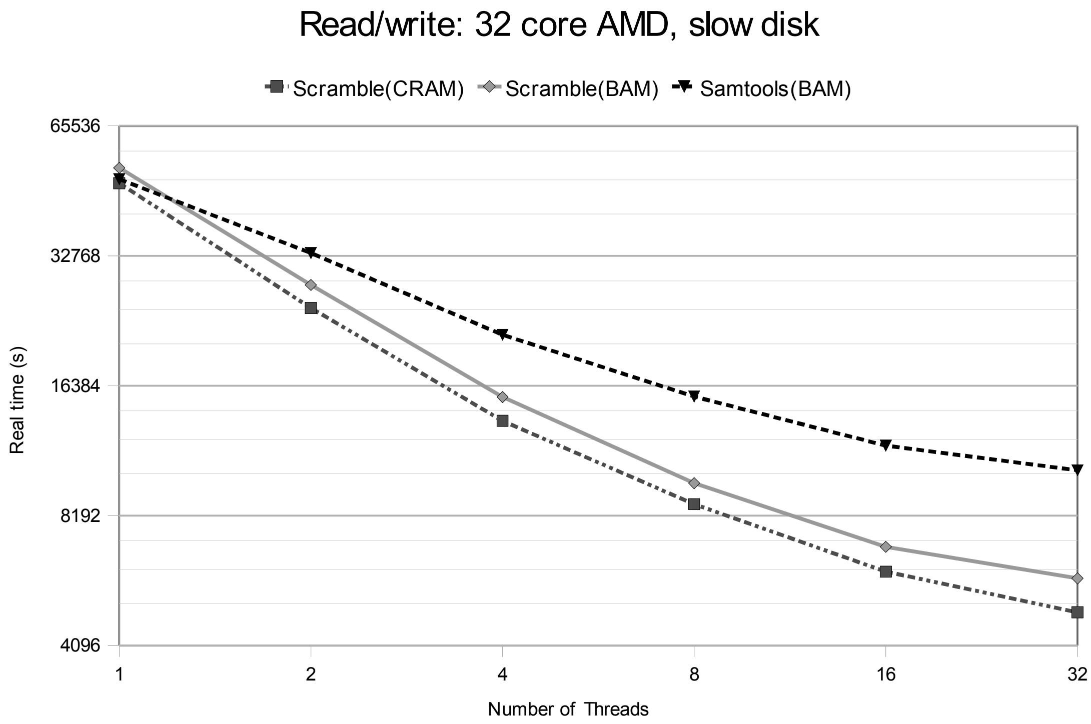


```
=== Scramble BAM->CRAM ===
32      R  4817.38      C 67378.62      S 2743.26
32      R  4959.48      C 67872.29      S 2706.82
16      R  5947.78      C 61252.62      S 2415.94
16      R  6201.05      C 61151.32      S 2379.59
8       R  8762.68      C 53405.49      S 2039.37
8       R  8653.06      C 53623.56      S 2052.74
4       R 13584.00      C 47973.65      S 1786.11
2       R 24799.04      C 47216.38      S 1739.68
1       R 48274.24      C 45421.60      S 1731.11

=== Scramble BAM->BAM ===
32      R  5871.00      C 87963.42      S 3447.51
32      R  5847.99      C 93327.67      S 3640.57
16      R  6922.95      C 72426.27      S 2722.93
16      R  6945.53      C 72343.54      S 2771.81
8       R  9914.64      C 60039.16      S 2452.21
8       R  9560.39      C 60314.78      S 2512.09
4       R 15419.40      C 52859.20      S 2574.91
2       R 28056.12      C 52390.66      S 2734.92
1       R 52382.03      C 49307.93      S 1317.87

=== Samtools BAM->BAM ===
32      R 10563.26      C 77712.68      S 3090.20
32      R 10305.17      C 77924.65      S 3115.85
16      R 12083.91      C 70362.21      S 2334.94
16      R 11713.07      C 70498.69      S 2348.70
8       R 15494.75      C 62713.28      S 1937.95
8       R 15421.81      C 62612.91      S 1943.83
4       R 21493.26      C 58898.60      S 1708.49
2       R 33254.78      C 55712.03      S 1563.66
1       R 49315.89      C 47773.25      S 1366.42
```

Read timings, fast disk I/O:

Tested on the 16 core 2.4GHz Intel Xeon system. This has superior local disk I/O performance, which is visible in the benchmarks for this large file. However it is still a realistic system given the nature of processing large data sets. We see no cross-over between BAM and CRAM speed here, but do see the saturation of disk I/O after 4 threads.

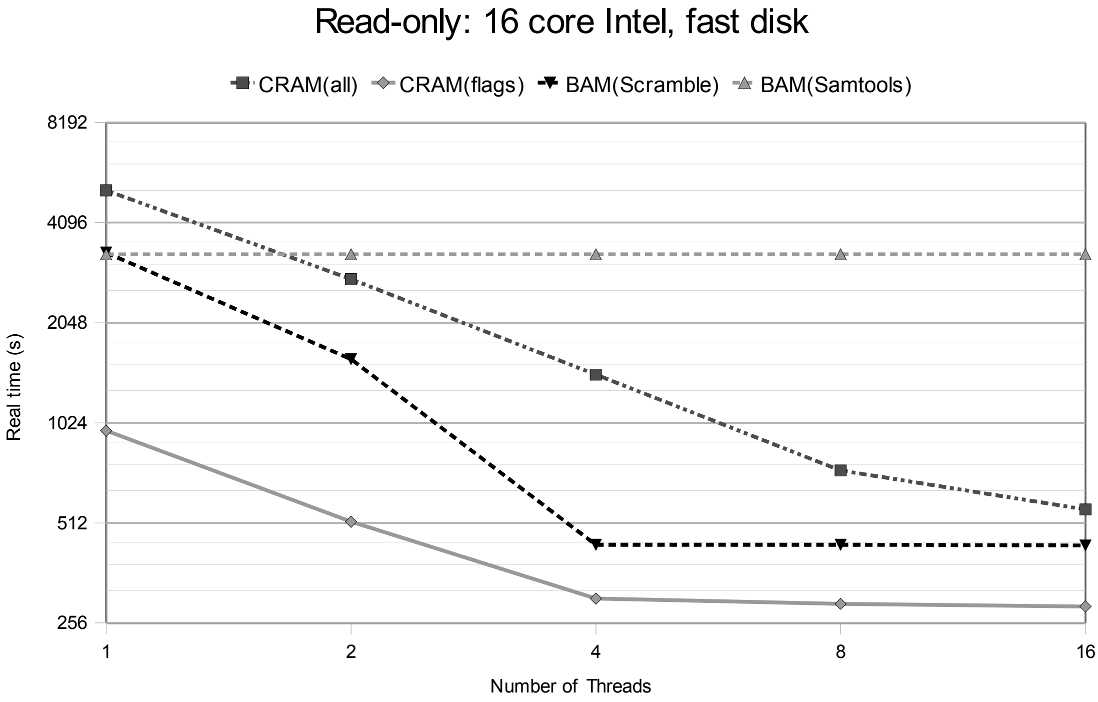


```
=== Scram_flagstat ===
16      CRAM(s) R 555.88       C 5681.71       S 285.40
16      BAM     R 439.75       C 4487.86       S 150.34
16      CRAM(f) R 288.56       C 864.11        S 191.05

16      CRAM(s) R 568.65       C 5674.75       S 284.60
16      BAM     R 437.95       C 4489.41       S 149.74
16      CRAM(f) R 286.23       C 865.60        S 190.50

8       CRAM(s) R 740.04       C 5472.69       S 764.76
8       BAM     R 440.62       C 3643.29       S 127.81
8       CRAM(f) R 292.39       C 861.02        S 207.99

8       CRAM(s) R 731.48       C 5500.69       S 703.03
8       BAM     R 439.88       C 3644.79       S 123.03
8       CRAM(f) R 292.04       C 867.24        S 202.75

4       CRAM(s) R 1429.89      C 5239.65       S 882.93
4       BAM     R 823.21       C 3486.83       S 140.89
4       CRAM(f) R 303.15       C 861.45        S 281.06

2       CRAM(s) R 2768.66      C 5078.85       S 883.04
2       BAM     R 1591.21      C 3405.12       S 142.98
2       CRAM(f) R 516.08       C 814.30        S 385.37

1       CRAM(s) R 5121.29      C 4941.37       S 143.91
1       BAM     R 3329.03      C 3169.35       S 122.15
1       CRAM(f) R 968.62       C 610.74        S 302.10

=== Samtools flagstat BAM ===
1       BAM     R 3289.66      C 3156.27       S 118.36
```

Read/write timings, fast disk I/O:

Scramble BAM → CRAM conversion scales very well here. Scramble BAM → BAM less so, possibly due to the larger files starting to have an impact on I/O performance.

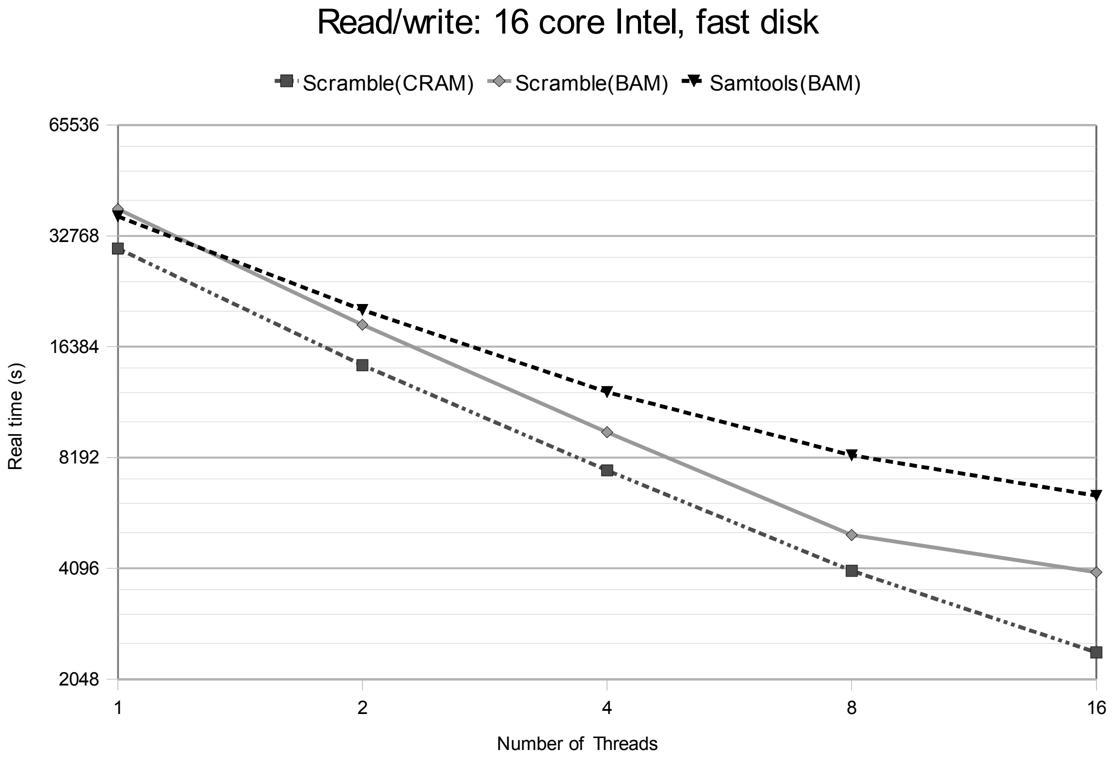


```
=== Scramble BAM->CRAM ===
16      R  2436.32      C 32242.52      S 1146.50
16      R  2406.51      C 32217.50      S 1136.25
 8      R  4000.53      C 30859.79      S 1027.65
 8      R  4068.51      C 30881.80      S 1006.09
 4      R  7557.97      C 29598.95      S  890.04
 2      R 14591.93      C 29091.76      S  866.78
 1      R 30278.08      C 29090.94      S 1062.98

=== Scramble BAM->BAM ===
16      R  4000.15      C 51016.51      S 1499.58
16      R  3996.70      C 51056.20      S 1462.96
 8      R  5043.17      C 39772.18      S 1065.15
 8      R  5043.98      C 39746.12      S 1052.43
 4      R  9599.08      C 38171.20      S  975.61
 2      R 18790.92      C 37435.52      S  881.66
 1      R 38751.41      C 37766.10      S  870.07

=== Samtools BAM->BAM ===
16      R  6438.96      C 40482.01      S 1185.10
16      R  6459.65      C 40481.46      S 1181.17
 8      R  8302.42      C 37960.42      S 1019.72
 8      R  8311.88      C 37970.61      S 1015.17
 4      R 12335.39      C 36653.49      S  909.65
 2      R 20625.44      C 36166.96      S  866.60
 1      R 37014.71      C 35986.91      S  945.21
```

Large file flagstat and index benchmarks

Test system: 16 core Intel machine with fast local disk. The disk cache was cleared before testing.

To evaluate the benefits of the CRAM format over BAM we also tried flagstat and indexing on the NA12878.mapped.ILLUMINA.bwa.CEU.high_coverage_pcr_free.20130906 data set. The flagstat test here is just a repeat of the single-threaded *scram_flagstat* and *samtools flagstat* commands in the above test.

Flagstat

```
$ sudo /usr/local/sbin/drop-cache
$ time scram_flagstat NA12878.mapped.ILLUMINA.bwa.CEU.high_coverage_pcr_free.20130906.cram
[flagstat output omitted]
real    15m23.472s
user    10m7.400s
sys     4m42.150s

$ sudo /usr/local/sbin/drop-cache
$  time samtools flagstat NA12878.mapped.ILLUMINA.bwa.CEU.high_coverage_pcr_free.20130906.bam
[flagstat output omitted]
real    54m25.951s
user    52m29.300s
sys     1m50.790s
```

Index

```
$ sudo /usr/local/sbin/drop-cache
$ time cram_index NA12878.mapped.ILLUMINA.bwa.CEU.high_coverage_pcr_free.20130906.cram
real    4m38.353s
user    0m8.730s
sys     2m22.700s

$ sudo /usr/local/sbin/drop-cache
$ time samtools index NA12878.mapped.ILLUMINA.bwa.CEU.high_coverage_pcr_free.20130906.bam
real    54m27.887s
user    52m38.630s
sys     1m42.940s

$ sudo /usr/local/sbin/drop-cache
$ time java -Xmx4000m -jar picard-tools-1.113/BuildBamIndex.jar VALIDATION_STRINGENCY=SILENT
VERBOSITY=ERROR QUIET=true I=NA12878.mapped.ILLUMINA.bwa.CEU.high_coverage_pcr_free.20130906.bam
real    70m58.223s
user    70m40.780s
sys     2m24.060s
```
